# Rehab-let: touchscreen tablet for self-training impaired dexterity post stroke: study protocol for a pilot randomized controlled trial

**DOI:** 10.1186/s13063-015-0796-9

**Published:** 2015-06-18

**Authors:** Debbie Rand, Gabi Zeilig, Rachel Kizony

**Affiliations:** Department of Occupational Therapy, School of Health Professions, Sackler Faculty of Medicine, Tel Aviv University, Tel Aviv, Israel; Department of Neurological Rehabilitation, The Chaim Sheba Medical Center at Tel-HaShomer, Tel-HaShomer, Israel; Sackler Faculty of Medicine, Tel Aviv University, Tel Aviv, Israel; Department of Occupational Therapy, University of Haifa, Haifa, Israel; Department of Occupational Therapy, The Chaim Sheba Medical Center at Tel Hashomer, Tel Hashomer, Israel

**Keywords:** Stroke rehabilitation, Upper extremity, Tablet, Games, Self-training

## Abstract

**Background:**

Impaired dexterity of the weaker upper extremity is common post stroke and it is recommended that these individuals practice many repetitions of movement to regain function. However, stroke rehabilitation methods do not achieve the required intensity to be effective. Touchscreen tablet technology may be used as a motivating tool for self-training impaired dexterity of the weaker upper extremity post stroke.

**Methods/Design:**

Rehab-let is a self-training protocol utilizing game apps on a touchscreen for practicing movement of the weaker upper extremity. We will conduct a pilot randomized controlled trial to assess Rehab-let compared to traditional self-training to improve dexterity of the weaker hand, and to increase self-training time and satisfaction in individuals with subacute stroke. Forty individuals with stroke undergoing subacute rehabilitation will be randomly allocated to Rehab-let or a traditional self-training program using therapeutic aids such as balls, blocks and pegs. All participants will be requested to perform self-training for 60 minutes a day, 5 times a week for 4 weeks. Dexterity assessed by The Nine Hole Peg Test is the main outcome measure. Assessments will be administered pre and post the self-training intervention by assessors blind to the group allocation.

**Discussion:**

The outcomes of this study will inform the design of a fully powered randomized controlled trial to evaluate the effectiveness of Rehab-let. If found to be effective, Rehab-let can be used during subacute rehabilitation to increase treatment intensity and improve dexterity. Potentially, Rehab-let can also be used after discharge and might be ideal for individuals with mild stroke who are often not referred to formal rehabilitation.

**Trial Registration:**

Current Controlled Trials NCT02136433 registered on 17 September 2014.

## Background

Weakness of the upper extremity is common post stroke, and a large population of individuals is left with a non-functional hand following their stroke [1, 2]. These Individuals need to practice many repetitions of movement at high intensity for efficient motor learning and neural plasticity to occur [3, 4]. However, despite the evidence, stroke rehabilitation does not achieve the required intensity [5–7]. Self-directed training, where the client practices the movements individually, is one way to increase training time and the number of repetitions. Individuals performing self-directed upper extremity exercises and activities, for an additional 30 minutes per day for 1 month during in-patient stroke rehabilitation, improved their functional ability and daily use of their weaker upper extremity compared to individuals who did not perform upper extremity self-directed training [8].

Games such as cards, pegs and blocks are commonly used in clinical practice to improve hand skills and potentially can be used for self-training. However, these traditional treatment methods are often boring and not motivating [9] and thus do not encourage intensive self-training in the clinic or at home. Moreover, with traditional therapeutic tools it is difficult to train speed of motion and reaction time and almost impossible to objectively quantify performance for precise and objective evaluation and as well as for monitoring improvement.

In the past decade commercial interactive video-games (e.g., EyeToy, Wii, Xbox Kinect) have been utilized by clinicians to motivate their clients in order to increase training time and promote health [10]. Despite the fact that video-games were not specifically developed for rehabilitation, they have been used in stroke rehabilitation to improve the function of the weaker upper extremity [11, 12] and to increase the repetition of purposeful movements [13]. However, video-games mainly encourage shoulder and elbow movements or gross hand movements, but not specifically dexterity (i.e., the ability to use the fingers in a coordinated and rapid manner). Dexterity is often impaired after stroke [14], and hinders the ability to use the weaker upper extremity for daily activities [15] such as eating or grooming.

Recent developments in touchscreen tablet technology may have the potential to fill this gap and serve as a self-training tool for finger dexterity [16]. These ubiquitous and portable computing devices with a web connection have a user interface that is built around a multi-touch screen. The hardware capabilities of these tablets include an accelerometer and a gyroscope that measure acceleration and orientation [17]. Application software, also known as an application or an app, is a computer software designed to help the user perform singular or multiple related specific tasks or games when using these devices. The user swipes, taps or pinches the screen with their fingers to perform game-like activities, which is more naturalistic than operating a mouse or using a computer keyboard. Many of the apps include gaming and music factors that appear to make a major contribution towards enhancing enjoyment and motivation. These factors have been found to facilitate participation in regular exercise [18].

Tablets have been recently used in pilot studies in rehabilitation. The iPad (Apple Inc., Cupertino, CA, USA) was perceived as a helpful tool for enhancing engagement in therapeutic activities and socialization [18] and found to have potential use for hand rehabilitation of individuals with stroke [19]. These preliminary findings support the potential use of tablets for stroke rehabilitation and the need for further research [18]. There are many health-related apps for tablets (e.g., to promote physical activity, monitor salt intake, weight loss); however, commercially available apps for rehabilitation of impaired dexterity are scarce and some of them are not released for public use. For example, Saposnik et al. [20] are currently examining their newly developed Stroke Rehab® game app for home-based rehabilitation poststroke. Countless game apps, which can be downloaded from the App Store (Apple Inc., Cupertino, CA, USA) or Google Market (Google, Mountain View, CA, USA), have been developed for the healthy general population. Despite the fact that they encourage finger and hand movement while playing fun games, only a limited number of apps might be successfully played by individuals with motor impairments due to stroke. Whereas the development of apps specifically for rehabilitation is warranted, it is unknown if existing tablet apps, that are accessible to everyone, can be used by individuals recovering from stroke with a mild to moderate motor upper extremity impairment. More so, due to the tablet's unique characteristics, it is unknown if playing these apps would result in improved dexterity, increased self-training time and higher client satisfaction compared to traditional directed self-training. We therefore propose a pilot randomized controlled trial (RCT) to compare the effectiveness of Rehab-let, a self-training protocol utilizing tablet technology with existing apps, to a traditional self-training program to improve impaired dexterity of individuals with stroke [8]. Individuals during subacute stroke rehabilitation will be targeted first since they receive on-going professional supervision. We hypothesize that using touchscreen tablets with existing, appropriately selected apps (Rehab-let), will result in improved dexterity, increased self-training time and high satisfaction in individuals with stroke as compared to the control group. These preliminary findings will assist with planning a future fully powered RCT.

### Trial design

A pilot single-blind randomized controlled study with assessments pre and post the intervention. Participants will be randomized by block randomization using an allocation ratio of 1:1 to receive self-training either by Rehab-let using the tablet or by performing traditional activities/exercises. All participants will perform the self-training in addition to the standard rehabilitation care.

## Methods

### Population

For individuals with stroke undergoing subacute in-patient rehabilitation to be eligible for this study, must comply with all of the following inclusion criteria:

1. Provision of written informed consent prior to entry into the study
2. Age above 20 years
3. At least 1 week post stroke
4. Evidence of ischemic or hemorrhagic stroke confirmed by computed tomography (CT) or magnetic resonance imaging (MRI) head scan
5. Full function of the hand prior to stroke
6. Ability to partially move their fingers
7. Cognitively intact or mild impairment (as assessed by a score above 20 on the Mini Mental State Examination [21])

Exclusion criteria: other neurological conditions besides stroke, uncontrolled seizures and acute orthopedic conditions of the upper extremity.

### Interventions

#### Rehab-let

A self-training protocol utilizing the Apple iPad touchscreen tablet (Apple Inc., Cupertino, CA, USA) with a variety of game-like apps will be used. We selected apps that encourage finger movement, could be motivating for adults, are not restricted in time and are not too fast. See Table 1 for examples of apps to be used.

**Table 1:** Examples of apps for self-training of experimental group

Name of app, developer, link	Screenshot	Description and aim
Dexteria BinaryLabs, Inc. http://goo.gl/b6rGE		Each finger needs to touch colored shapes in a timely manner/trace letters/pinch crabs walking on the screen. Encourages finger dexterity and isolation of movements, and provides a performance report. It was developed specifically for training fine motor skills, but not purposefully for stroke
FastTouch Pedro Riera http://goo.gl/6yfZlq		Finger tapping as many times as possible in a pre-fixed time period. Encourages speed of movement and can be performed with one or more fingers
Scribble Kid Mile 26 Studios http://goo.gl/OdwDce		Tracing and drawing on the tablet with fingers or with a stylus. Encourages accuracy and qualitative movement of the hand
Fruit Ninja Halfbrick Studios http://goo.gl/TkaAS		Cutting (swiping) fruit that appear in different locations on the screen, in increased frequency while avoiding other shapes. Encourages eye-hand coordination, reaction time and accuracy. Recommend to play "Arcade" mode within a time frame

#### Traditional self-training

The GRASP (Graded Repetitive Arm Supplementary Program) [8], which is a self-directed arm and hand exercise program developed for individuals with stroke with upper extremity impairments, will be used. It is comprised of instruction manuals suited to the functional level of the upper extremity and an equipment kit (such as a towel, a ball and pegs). This self-training intervention has been proved to be more efficient than an educational program for improving the weaker upper extremity post stroke [8] and can be freely downloaded from the Internet (http://neurorehab.med.ubc.ca/grasp/). More so, the GRASP has been implemented in many stroke rehabilitation centers as a cost-effective way to improve the function of the weak upper extremity [22].

### Implementation of the intervention

Individuals will be approached during their first week in rehabilitation and their written consent will be obtained. Following the first assessment, individuals will be randomly allocated to receive self-training either by Rehab-let (experimental group) or GRASP (control group). Concealed allocation will be maintained. A single session will be provided to each participant by an occupational therapist (OT) to teach them how to play the apps (experimental) and perform the exercises (control). Each participant will also receive a booklet with explanations and photographs of the self-training apps/exercises and a log sheet. Participants will be requested to fill in a dailylog regarding their training time. In addition, participants will be asked to indicate whether they have had assistance or someone present during the self-training. Participants will be instructed to play the apps (experimental)/perform the exercises (control) with their weaker upper extremity for a total of 60 minutes a day for 5 days over a 1-month period. The 60 minutes can be divided into 2 or 3 daily sessions. The OT will monitor the participants weekly to assure training is been performed daily, to verify logs are filled in accurately, to adapt the level of the apps/exercises if needed, and to assure that there are no adverse effects. Participants will be given the OT's phone number and will be encouraged to contact him/her during the week, if needed. Participants randomized to the Rehab-let self-training will be taught how to play 4–5 different apps on the tablet, selected together with the OT from a pool of 20 apps. These apps will be available for them in a folder on the iPad’s screen. See Table 2 for a summary of the study’s interventions.

**Table 2:** Description of study interventions based on the intervention description and replication (TIDieR) template [38]

Brief name	Rehab-let
Why	Increasing treatment intensity can improve the impaired dexterity of the weaker upper extremity however there is insufficient training time for the weak upper extremity during subacute stroke rehabilitation
What	a. Existing iPad Apps (from the Apple Store (Apple Inc., Cupertino, CA, USA)) using the Rehab-let protocol (experimental), traditional treatment aids such as blocks, pegs and a towel using the GRASP protocol (control)
	b. A printed manual for self-training of hand movements with description and photos of the apps (experimental) or of the exercises (control)
Who provides	Intervention (supervision) and assessments will be performed by occupational therapists who are experts in neurological rehabilitation
How	The intervention will include self-training during inpatient rehabilitation. Face- to-face individual follow-up meetings with the occupational therapists will occur once a week
Where	Intervention will take place in in-patient rehabilitation departments. Participants can self-train either in their room or in a quiet corner in the department
When and how much	Individuals will be requested to self-train 60 minutes a day, 5 times per week for 4 weeks (total of 20 sessions). Four meetings with the occupational therapist will be held during this time as well as pre and post assessment sessions
Tailoring	Different apps from a pool of about 20 apps that facilitate finger and hand movement (experimental) or different exercises from the GRASP protocol (control) will be taught to each participant according to the motor ability of their upper extremity and preference

#### Outcome measures

The primary outcome measure is improved dexterity, which will be assessed by The Nine Hole Peg Test (NHPT) [23]. This test is a valid and reliable test to assess dexterity in individuals with stroke [24] and the time to insert and remove 9 pegs is measured. The following secondary outcome measures will be used; grip and pinch strength [25] will be assessed by the Jamar and pinch dynamometer. The Fugl-Meyer Motor Assessment [26] will assess the motor impairment of the upper extremity. The Action Research Arm Test [27] will assess the functional ability of the upper extremity by grasping and moving objects of different size and weight. The Visual Analogue Scale [28], which quantifies the perceived level of pain intensity on a scale of 0–10 cm, will also be used. Self-training time will be used to assess adherence to the self-training protocol and will be taken from the daily logs. Satisfaction from the self-training program will be determined by a Satisfaction Questionnaire, developed for the study. The questionnaire will query the participant's satisfaction with the self-training intervention and his or her perceived benefit of the intervention to improve upper extremity’s function. Responses will be rated on a scale of 1–5. The System Usability Scale [29] will be filled in by individuals in the Rehab-let group – to assess their subjective usability of the tablet for rehabilitation. Each of the 10 items is rated on a 5-point scale from 1 (disagree totally) to 5 (agree totally), an overall score is calculated ranging from 10 (low usability) to 100 (high usability) points. Demographic and stroke characteristics will also be collected at baseline as well as information regarding prior tablet experience. In addition, independence in basic activities of daily living of this population will be characterized using the Functional Independence Measure [30].

#### Data collection

**Fig. 1:**
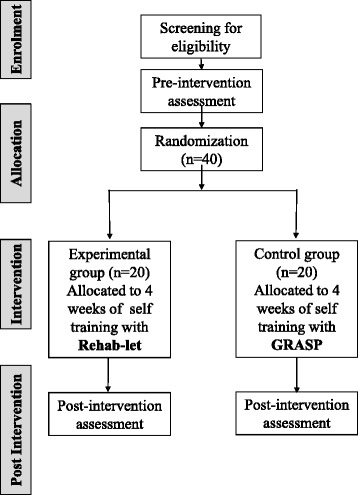
Study flow design

The clinical measures mentioned above will be administered pre and post the intervention to test the effectiveness of the intervention. See Fig. 1 for study flow.

#### Blinding

Assessments pre and post the 4-week self-training intervention will be administered by assessors blinded to the group allocation. See Fig. 1 for study flow.

#### Study samples

A total of forty participants will be recruited for this pilot study: 20 randomized to the Rehab-let self-training protocol (experimental group) and 20 to the GRASP self-directed training (control group). These results will be sufficient to calculate the effect size to use in sample size power calculations for a larger RCT.

### Data analysis plan

All data will be inputted in IBM SPSS statistics software, version 21 (Armonk, NY, USA). Descriptive statistics for the study population and outcome measures will be computed for both groups to verify that groups are similar preintervention. A repeated measures analysis of variance (ANOVA) will be used to compare the NHPT scores within and between groups; with repeated measures for time (pre intervention versus post intervention) between the groups (Rehab-let versus GRASP) and for interaction effect between time and group. A difference above 32.8 seconds [31] on the NHPT for the weaker upper extremity will be considered to be a clinically meaningful change. Differences in the upper extremity secondary measures as well as adherence (self-training time) and satisfaction will be compared as well. Intention-to-treat analysis will be conducted with the last observation carried forward method.

### Privacy, safety, and ethics

We have no safety or ethical concerns regarding this trial. Individuals will consent prior to enrollment. We already have an approval (#0125-13-SMC) from the Sheba Medical Center Institutional Helsinki Committee. Individuals will be encouraged to report adverse effects such as pain and, when necessary, the study will be stopped. Participants from both self-training interventions may potentially improve their dexterity and, therefore, benefit from the program.

## Discussion

Self-training is a time-efficient and cost-effective way [8] to increase treatment time for the weaker upper extremity. Since clients are engaged in treatment sessions for a very limited time during each day in in-patient rehabilitation, and may experience boredom and loneliness [6, 7], self-training can be utilized during subacute rehabilitation as well as at home following discharge. We have developed Rehab-let, a self-training program, by utilizing generic touchscreen tablets’ apps. Many adults already use tablets for their everyday life, social interactions and for playing games. Utilizing the tablet with game-like apps is inexpensive, accessible and may be motivating for clients to perform the countless repetitions needed for effective brain plasticity to occur [3, 4]. If Rehab-let is proven to be an effective tool for improving the upper extremity function, particularly dexterity, enhancing adherence to the self-training protocol (self-training time) and satisfaction, then we might have identified an exciting and motivating tool for stroke rehabilitation that will need to be further examined to establish evidence-based practice.

As opposed to commercial video-games, there are minimal risks for self-training impaired dexterity with tablets, so the benefit for the clients might be more significant. In the future, tablets may be used to train other impairments, such as cognitive or language deficits, which are common post stroke [32]. Moreover, such a self-training protocol may help transfer some of the responsibility of rehabilitation to the clients themselves: i.e., self-management, thus possibly increasing their self-efficacy as well as improvement of the weaker upper extremity. Further research should determine this.

Once the effectiveness of Rehab-let is established, it can be implemented in the home setting for individuals discharged from rehabilitation with a mild to moderate weaker upper extremity. Rehab-let may actually be most suitable for individuals with mild stroke. Mild stroke accounts for 60 % of all cases of stroke [33] and, despite the fact that these individuals are considered not to have disabling functional deficits [34], they might experience motor, sensory, visual, speech or cognitive deficits [34, 35]. These individuals are usually discharged home [36] without further rehabilitation since they are expected to return to their premorbid status [37].

To summarize, our findings will help design a fully powered RCT to determine the effectiveness of Rehab-let for improving impaired dexterity of the weaker upper extremity post stroke. More so, we will be able to determine the profile of individuals who can benefit from this protocol and to provide guidelines for developing apps that are tailored for people with stroke who cannot benefit from existing apps.

### Trial status

Currently recruiting participants.

## Abbreviations

ANOVA: analysis of variance
app: application software
CT: computed tomography
GRASP: Graded Repetitive Arm Supplementary Program
MRI: magnetic resonance imaging
NHPT: Nine Hole Peg Test
OT: occupational therapist
RCT: randomized controlled trial
Rehab-let: self-training protocol utilizing game apps on a touchscreen for practicing movement of the weaker upper extremity.
